# Hepatitis C Viral Heterogeneity Based on Core Gene and an Attempt to Design Small Interfering RNA Against Strains Resistant to Interferon in Rawalpindi, Pakistan

**DOI:** 10.5812/hepatmon.6184

**Published:** 2012-06-30

**Authors:** Sobia Kanwal, Tariq Mahmood

**Affiliations:** 1 Faculty of Biological Sciences, Quaid-i-Azam University, Islamabad, Pakistan

**Keywords:** Interferons, Small interfering RNA (siRNA), Hepatitis C

## Abstract

**Background:**

Global prevalence of Hepatitis C Virus (HCV) infection corresponds to about 130 million HCV positive patients worldwide. The only drug that effectively reduces viral load is interferon-α (IFN-α) and currently combination of IFN and ribavirin is the choice for treatment.

**Objectives:**

The present study is aimed to resolve the genotypes based on core gene that might affect the response to interferon therapy. Furthermore an attempt was made to propose a powerful therapeutic approach by designing the siRNA from sequences of the same patients who remain resistant to IFN in this study.

**Patients and Methods:**

To achieve the objectives, a sequence analysis was performed in five HCV ELISA positive subjects who have completed IFN treatment. Neighbor Joining (NJ) method was used to study the evolutionary relationship. Atomic models were predicted using online software PROCHECK and i- TASSER.

**Results:**

Two new genotypes were reported for the first time namely 4a from suburban region of Rawalpindi and 6e from all over the Pakistan. According to Ramachandran plot, satisfactory atomic model was considered useful for further studies, i.e. to calculate HCV genotypes conservation at structural level, to find out critical binding sites for drug designing, or to silence those binding sites by using appropriate siRNA. Single siRNA can be used to inhibit HCV RNA synthesis against genotype 3 and 4, as the predicted siRNA were originated from the same domain in studied HCV core region in both genotypes.

**Conclusions:**

We can conclude that any change or mutation in core region might be the cause of HCV strains to resist against IFN therapy. Therefore, further understanding of the complex mechanism involved in disrupting viral response to therapy would facilitate the development of more effective therapeutic regimens. Additionally, a single designed siRNA can be used as an alternative for current therapy against more than one resistant HCV genotypes.

## 1. Background

Hepatitis C virus (HCV) is endemic worldwide infection and its distribution varies geographically. Prevalence estimate is much higher among developing countries than in developed countries [1]. Pakistan holds one of the world’s largest burdens of chronic hepatitis disease and death incidence due to liver failure and hepatocellular carcinoma [2]. More than 10 million people that make up to 6 % of total population of Pakistan are suffering from HCV, with high morbidity and mortality [3]

Phylogenetic analyses identified six major lineages, namely genotype 1-6. These groups were further subdivided into several subtypes. The creation of quasispecies is possible due to high rate of mutation in hepatitis virus strains, even within a single infected individual. On the basis of genetic similarities, numerous viral strains have been categorized into groups, types, and subtypes. Genotype 1 is the most common lineage in North and South America as well as in Europe [4]. However, distribution of the genotypes in Asia- Pacific region is diverse. In contrast to Japan and China where the predominant genotype is 1, and the Middle East where the major genotype is 4, genotype 3 is common in Pakistan and is detected in 67-87 % cases [5]. The interesting fact about genotype 3 is that besides Pakistan, India, and Bangladesh, it is also a major genotype in Australia and New Zealand. Furthermore, in the context of Pakistan, Idress et al. [3] have reported that genotype 3a is the most common type in all provinces except in Baluchistan where the most popular subtype is 1a. HCV is endemic worldwide and its distribution varies geographically. Reported HCV prevalence in Pakistan is much higher when compared with other countries of the region like India (0.9 %), Indonesia (2.1 %), and China (3.2 %) [6]. Therapeutic approaches against HCV include antiretroviral treatment, inhibitors, RNAi, or siRNA. Interferon (IFN) alpha, a naturally occurring cytokine that increases the immune response against HCV is considered the only therapy for chronic hepatitis C; injected PEG-IFN is hypothesized to function by mimicking this natural cytokine [7]. Another antiviral agent ribavirin (RBV) is devoid of considerable antiviral activity when used alone in HCV infection [5] but it significantly increases the antiviral effect of IFN when used in combination treatment [8]. Various HCV genotypes respond to interferon in different ways [9]. HCV genotype 1b (HCV1b) is resistant to interferon with lesser response of only 10-40% as compared to other genotypes like HCV-2a or HCV-2b, that showed complete response of 60-90 % [10][11]. HCV-1b is the most recurrent variant worldwide, with a high occurrence (37-80 %) in Asian, American, and European countries. Patients infected by HCV-1b genotype suffer from a more active disease and are more prone to liver cirrhosis and hepatocellular carcinoma than patients with other HCV genotypes [12]. Amantadine is another antiviral agent that reduces viral replication by interfering with virus uncoating or transcription of viral RNA. Moreover, a major research struggles to develop ‘Specifically Targeted Antiviral Therapy for HCV’ (STATC). The best knowledge about molecular structure of HCV, its proteins components, and diverse stages of replication cycle of the virus, specific small molecules, lead to development of viral enzymes inhibitors. Some new antiviral drugs include Telaprevir, Boceprevir, Danoprevir, Nucleoside analogues, nucleotide analogue, non-nucleoside analogue, caspase inhibitors, and cyclophillin inhibitors [13]. Drugs that are under the development include small molecules such as protease inhibitor, polymerase inhibitor, and toll-like receptor drug classes. While many of these drugs seem to hold promise as either a primary or an adjunctive treatment for patients with chronic hepatitis C, they are years from market and their safety and efficacy are uncertain in difficult-to-treat patients [14].

Practice of siRNA is more valuable as it binds directly to specific mRNA results in exclusive block of transcription potentially bearing a powerful molecular therapeutic approach rather than current therapy of highly liable to RNAi-induced suppression, as the inhibition of HCV RNA levels by targeting different genes using RNAi has been reported [15]. HCV Core protein is involved in a whole array of host cell functions including signal transduction, and transcriptional regulation of genes in the liver. Many reports showed that substitutions in HCV core region consequences enhanced insulin resistance, liver steatosis, oxidative stress and Hepatocellular Carcinoma (HCC) [16]. Current therapies against HCV demonstrate limited efficiency due to development of viral resistance and high rate of HCV mutation. The problem of viral mutants could be resolved by using a mixture of siRNAs against different sequences. Several studies have also revealed the feasibility of targeting host cellular and viral factors involved in HCV infection as potential therapeutic goals [17][18].

## 2. Objectives

In the present study the genotypes of five HCV positive but unresponsive to IFN therapy were resolved. Further, an attempt was performed to design an anti-HCV siRNA in studied samples to be employed against any HCV genotype.

## 3. Patients and Methods

### 3.1. Patient and Sample Selection

HCV Enzyme linked immune sorbent assay (ELISA) positive individuals who have completed IFN treatment were randomly selected from Rawalpindi General Hospital. Blood samples were collected from patients after taking informed written consent.

### 3.2. RNA Extraction, cDNA Synthesis, and Amplification of Core Region

Qualitative detection of HCV RNA was performed using Reverse transcriptase (RT) PCR. Briefly, 150 μl of patients’ sera samples were used to isolate the RNA by a commercially available kit (NucleoSpin RNA Virus by Machereynagel) according to the manufacturer’s instructions. Complimentary DNA (cDNA) of partial core region HCV was synthesized using 100 units of Moloney murine leukemia virus (MMLV) reverse transcriptase enzyme (Fermentas, USA) with 10 μM of outer antisense primer. Two PCR amplification cycles were performed (first cycle PCR and nested PCR) with five units of Taq DNA polymerase enzyme (Fermentas, USA) in a volume of 25 μl reaction mixture. External PCR conditions performed in a thermal cycler were as follows: an initial denaturation step at 95°C for 3 minutes followed by 30 cycles of 94°C for 30 seconds, 55°C for 30 seconds, 72°C for 1 minute, and finally extended at 72°C for 3 minutes. Internal PCR conditions were the same except for a different set of inner primers used with annealing temperature for 5 minutes in cycle 1 of PCR amplification. Products of nested PCR were directly sequenced on Beckman Coulter CEQ 8000 sequencer after purification by PCR Product Purification Kit from Genomed.

### 3.3. Phylogenetic Analysis

Five obtained sequences were aligned by ClustalW and similarity of sequences with which already reported in database (http://blast.ncbi.nlm.nih.gov/Blast.cgi) was found by Nucleotide Blast (nBlast). [19]. After application of Tajima’s test [20] and Neighbor Joining (NJ) methods, obtained statistical selection pairingwas used for phylogenetic tree construction [21]. Base statistical robustness was performed by 500 bootstrap repeats and the whole process was developed by MEGA 5 software [22].

### 3.4. Protein Structure and Function Prediction

Protein structure and function was predicted using i-TASSER server after translating the nucleotide sequence into amino acid. 3D models were built based on multiple-threading alignments and i-TASSER assembly simulations; function insights were then derived by matching the predicted models with protein function database [23][24].

### 3.5. Stereo Chemical Evaluation of 3D Protein Models

3D structural models built using i-TASSER were evaluated on PROCHECK. It checks the stereochemical quality of a protein structure that produces a number of plots in PostScript format analyzing its overall and residue-by-residue geometry. Pdb files of 3D models were uploaded and Ramachandran plot was used to check the existence of five models predicted by i-TASSER for each sequence.

### 3.6. siRNA Prediction for HCV

Antiviral siRNA prediction was made for HCV core region. SiRNA sequences were selected based on their degree of conservation, defined as the proportion of viral sequences that were targeted with complete matches by corresponding siRNA. SiDirect was employed to predict siRNA as a highly effective, target specific siRNA online design tool. Five sequenced HCV samples were pasted in FASTA format and the program was run by implementing Ui-Tei algorithms, the algorithms that are on the back hand of siDirect [25]. The output page displays siRNA sequence and siRNA position.

## 4. Results

Partial sequencing of core region of five HCV positive samples resistant to IFN therapy (Pak-01-10, Pak-02-10, Pak-03-10, Pak-04-10, Pak-05-10) revealed different genotypes mentioned in Table 1.Genotype 3 (3a and 3b) was resolved by samples Pak-0110, Pak-04-10, and Pak-05-10 with high percentage similar to already reported sequences. Genotype 4a was observed by sample Pak-02-10 while nBlast resolved sample Pak-0310 of genotype 6e. Genotype 6e was reported for the first time in Rawalpindi, Pakistan.

**Table 1:** Percentage Similarity of Sequenced Samples With Reference Sequences From nBlast

	**Accession Number of Reference Sequences**	**Similarity, %**	**Genotype**
Pak-01-10	AB523124	98	3b
Pak-02-10	DQ988076	96	4a
Pak-03-10	AB301826	98	6e
Pak-04-10	DQ777803	93	3b
Pak-05-10	EU81436	97	3a

### 4.1. Phylogenetic Analysis

**Figure 1:**
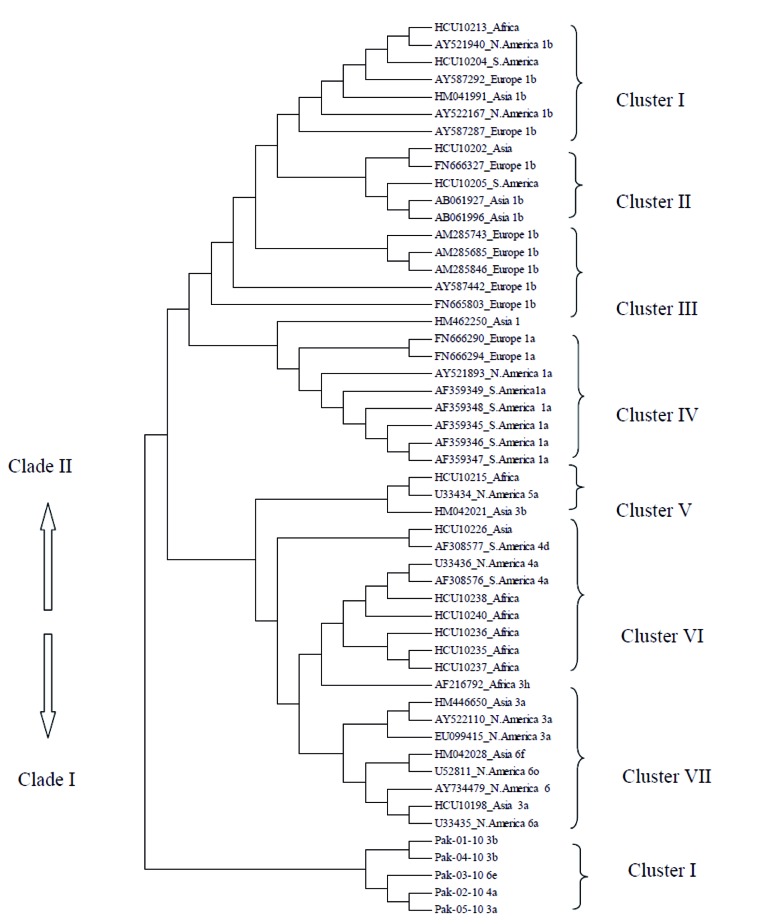
Phylogenetic Tree of Studied HCV Sample Sequences of Core Region and Reference Sequences Using Pair Wise Alignment Tool of ClustalW in Online Available Software Mega 5. The reference sequences are indicated by accession numbers and samples of the present study are indicated by Pak-01-10, Pak-02-10, Pak-03-10, Pak-04-10, and Pak-05-10.

Aligned sequences using multiple alignment software Clustal W were further subjected to construct tree using the software MEGA 5 to find out the relationship between the sequences, novel genotypes, subtypes, and variants. Unrooted NJ tree constructed for studied samples with reference sequences (Africa, Europe, Asia, North America, and South America) from Los Alamos mounted from two main clades that show the presence of separate genetic lineages. Clade 1 showed all samples of the present study while clade 2 exhibited irregular pattern of evolution among studied samples representing different geographical regions worldwide however separated in different clusters (Figure 1). Active rate of mutation in HCV core region was shown by tree topology in all different geographical regions i.e. European strains (clade 2, cluster I, II, and III), North American and South American strains (clade 2, cluster III). Sequences from Asia appeared at distinct positions in a tree showing high level of diversity (clade 2, cluster I, II, V, VI) (Figure 1). In clade 1, single cluster showed a clear independent clustering of studied sequenced samples. Clade1 has shown active and early branching pattern of subtype 3b in Pakistani population depicted that genotype 3 was actively evolving in IFN resistant strains of HCV in inhabitants of Pakistan.

### 4.2. 3D Structural Analysis

**Figure 2:**
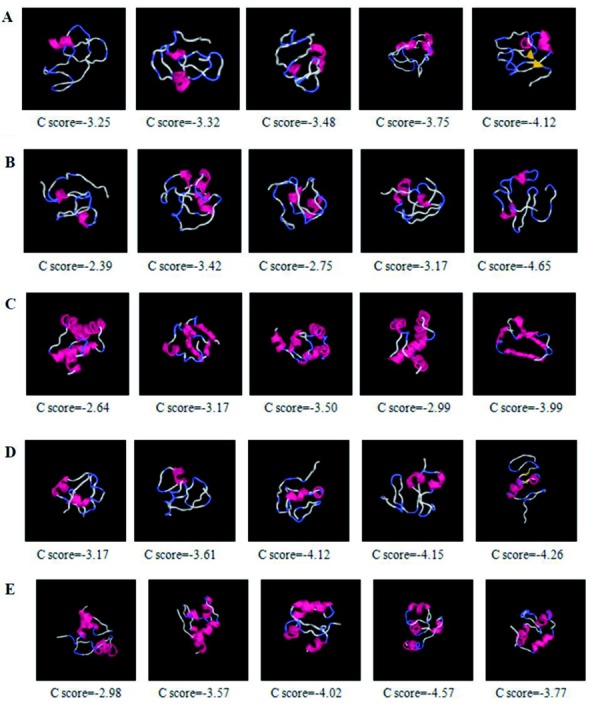
Predicted 3D Protein Models Built by i-TASSER for the Sequence. (A) Pak-01-10; (B) Pak-02-10; (C) Pak-03-10; (D) Pak-04-10; (E) Pak-05-10 C-score from i-TASSER that reveals the strength of the predicted model. The model with the highest score (first one) is the most likely to exist.

**Figure 3:**
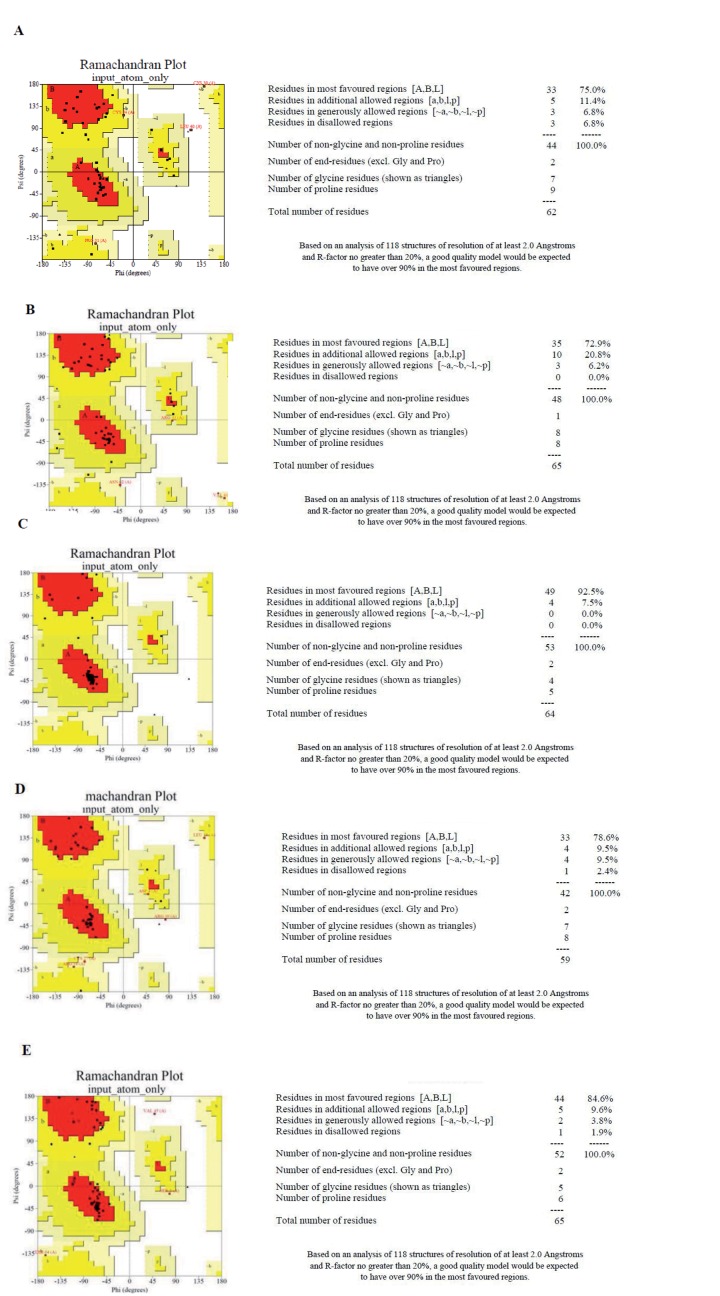
Ramachandran Plots Constructed Using PROCHECK for Evaluation of 3D Structural Models Predicted With the Help of i-TASSER. These PROCHECK builds of protein models demonstrate whether the amino acid residues exist in the “favored region” or “disallowed region” of the plot. For a good protein model there must be ≥ 90 % residues in the most favored region or < 2 % in the disallowed region of the plot. Ramachandran plots showing the residues in most favored and disallowed regions for sample: (A) Pak-01-10, (B) Pak-02-10, (C) Pak-03-10, (D) Pak-04-10, (E) Pak-05-10

Models for the studied sequences were first built by the use of i-TASSER which generates up to five full-length atomic models (ranked based on cluster density) of the studied samples with resolved genotypes. Ramachandran plots were built using PROCHECK to evaluate these models for 3D structural analysis (Figure 2 A-E). The next step was to determine the most likely to exist among five predicted atomic models by using a software for model evaluation. Ramachandran plots were built through PRO-CHECK to evaluate the 3D structural models predicted by i-TASSER. PROCHECK builds of protein models show whether the amino acid residues lie in the “favored region” or “disallowed region” of the plot. For a good protein model, there must be ≥ 90 % residues in the most favored region or < 2% in the disallowed region of the plot. Considering the Ramachandran plots model 1 of Pak-03-10 was almost well for further analysis (Figure 3C) as about 90 % (88 %) residues were found to be in the most favored regions, while less than 2 % (1.1 %) were in the disallowed region. Analysis of the Ramachandran plots for the remaining four models revealed that only model 1 should be analyzed further. Similarly, model 4 of Pak-01-10 (Figure 3A), model 4 of Pak-02-10 (Figure 3B), model 5 of Pak-04-10 (Figure 3D), and model 3 of Pak-05-10 (Figure 3E) were considered satisfactory (Figure 3B to 3G). Detailed score of Ramachandran plot for all five sequences is given in Table 2.

Models predicted by i-TASSER with highest C-score as well as satisfactory plot statistics (< 75 %) can be further used for 3D models analysis which could help us to predict the cleavage sites or recognition of phosphorylation sites.

**Table 2:** Ramachandaran Scores of Models for All the Five Sequences Predicted by PROCHECK

	**Model 1, %**	**Model 2, %**	**Model 3, %**	**Model 4, %**	**Model 5, %**
Pak-01-10					
Allowed	65.9	72.7	72.7	75	72.7
Disallowed	2.3	9.1	0	6.8	4.5
Pak-02-10					
Allowed	60.4	66.7	56.2	72.9	66.7
Disallowed	4.2	6.2	2.1	0	0
Pak-03-10					
Allowed	92.5	49.1	83.0	88.7	28.3
Disallowed	0	7.5	1.9	1.9	3.8
Pak-04-10					
Allowed	69	61.9	61.9	52.4	78.6
Disallowed	2.4	2.4	4.8	7.1	2.4
Pak-05-10					
Allowed	80.8	80.8	61.9	52.4	78.6
Disallowed	0	3.8	4.8	7.1	2.4

### 4.3. Tajima’s Test of Neutrality

Tajima’s test of neutrality collates the number of discriminating sites per site with the nucleotide assortment. A site is considered segregating if in a comparison of sequences, there are two or more nucleotides at that site. The Tajima test was calculated using MEGA 5. All gaps were eliminated from the data group; m = number of sites, S = number of segregation sites, Ps = S/m, θ = ps/a1, and л= nucleotide diversity. D is the statistical test result.

### 4.4. Prediction of siRNA for HCV Core

Highly effective siRNA sequences were selected by using novel guidelines that were established through an extensive study of the relationship between siRNA sequences and RNAi activity by online available software siDirect. The siRNA was predicted against five HCV samples that were resolved in the present study (Table 3). The predicted siRNA from same domain of core region showed that RNA and DNA binding domain was conserved in core region in genotype 3 and 4; so single siRNA can be used against both genotypes and inhibit HCV RNA synthesis.

**Table 3:** Predicted siRNA Against the Present Studied Resolved HCV Samples

	**HCV genotype**	**Target Position**	**Target Sequence**	**siRNA sequence**
Pk-01-10	3b	24-46	CCGCTCAATACCCGGAAATTTGG	AAAUUUCCGGGUAUUGAGCGGGCUCAAUACCCGGAAAUUUGG
Pak-02-10	4a	26-48	CCGCTCAATGCCCGGAAATTTGG	AAAUUUCCGGGCAUUGAGCGGGCUCAAUGCCCGGAAAUUUGG
Pak-03-10	6e	155-177	TGCACAAGTTCGTGACTTTTAAA	UAAAAGUCACGAACUUGUGCACACAAGUUCGUGACUUUUAAA
Pak-04-10	3b	25-47	CCGCTCAATACCCGGAAATTTGG	AAAUUUCCGGGUAUUGAGCGGGCUCAAUACCCGGAAAUUUGG
Pak-05-10	3a	24-46	CCGCTCAATACCCAGAAATTTGG	AAAUUUCUGGGUAUUGAGCGGGCUCAAUACCCAGAAAUUUGG

## 5. Discussion

Genotype is one of the strongest predictive aspects of sustained virological response [26]. In the present study four different genotypes have been revealed among five HCV positive samples resistant to IFN while two samples remained without recognized genotypes. Genotype 3 is prevalent is Pakistan [27][28] and the increase in the number of genotype 3 (a and b) patients along the time in Pakistani population have been observed by various scientists [29][30]. Present study demonstrates that genotype 3 (a and b), 4a, and genotype 6e do not respond to IFN therapy. Genotype 4a is reported for the first time in this study from suburban areas of Rawalpindi by sample Pak-02-10. This is the most important and prevalent strain of Egypt [31], North Africa and the Middle East [32][33]. Iqbal et al. [26] and Idrees et al. [3] reported genotype 4 in the blood samples of Pakistani population from other geographical regions of the country. The possible existence of this genotype in Pakistan might be due to the prevalence of genotype 4 in neighboring country Iran with geographical location near to Europe and Middle East [29][30]. Genotype 4 has been reported to be frequently coupled with severe cirrhosis and a reduced response to interferon therapy [34][35]. Similar to Pakistan, HCV genotype 4 is also infrequent in the United Sates and there are few published data regarding response to therapy in patients with HCV genotype 4 infections in both Pakistan and United States [36]. Genotype 6e is reported for the first time in Rawalpindi, Pakistan by the sample name Pak-03-10. This genotype 6 is frequent amongst patients from Southeast Asia [32][33]. An earlier study found that genotype 6 was spread widely through Southeast Asia and was not limited to injection drug users [37]. Rare genotypes reported from Pakistan include 1c, 1d, 2c, 2k, 3c, 3k, 4, 5a, 6a, and 6v [3][38]. It is quite promising that these two new genotypes may have entered into Pakistan from other countries through local persons who cross borders for job and trade. Shift in HCV genotype circulation needs to be paid more consideration. This enhances an alarming signal on the major steps to be taken to reduce such infection as this genotype is correlated with severe cirrhosis. It has been reported that failure to typing a genotype is caused by mutations [39][40] or may be insertions, deletions, or inversions and translocations. HCV do not perform proof reading and its high mutation rate made it genetically successful according to Darwinian Theory of natural selection [41]. So, these two samples that failed for typing in the present study might be either a novel variant of the existing genotype or representative of the recombinant forms of the mixed genotype. Idrees et al. [3], Afridi et al. [42] reported 14 %, 32.14 % and 14.13 % novel genotypes in Lahore and Quetta cities of Pakistan, respectively. Therefore, on the basis of these facts it can be concluded that genotype distribution is not even in all areas of Pakistan. The rate of distribution of genotypes and their genetic makeup varies at sub population levels of same area.

### 5.1. Phylogenetic Analysis

The NJ tree was constructed for the studied samples with reference sequences from the (LANL) mounted by two main clades showed the presence of separate genetic lineage. The Dendrogram was developed to find an association between studied samples and reference accessions from other countries; overall, it was revealed that the studied HCV samples of the present study exhibited long branch lengths, indicating an ancient history of their evolution and their genetically stable genome composition; this might be attributed to the suppressed or compromised immune pressure of the host. Previous reports supported the evidence that cases where immune response is compromised, there are a less chance of viral clearance [43][44]. Secondly, it was evident that history of evolution of virus is more ancient in Pakistan than other countries. on the other hand, the accessions from world over are actively mutating and more divergent, or it can be said that they are still in an active phase of evolution. Moreover, it is evident from the dendrograms that disease is endemic in Pakistan for more time period than reference countries as it is already established that HCV prevalence in Europe is not homologous with reference to the distribution of genotypes [45][46]. Pybus et al. [37] particularly with reference to Asia, explained the origin and maintenance of HCV diversity and reported that Asian model of evolution could be a baseline for investigating HCV spread in other continents. Similarity of Asian strains with all the reference strains is might be due to some migration events. It demonstrates the relationship with European strains; a study described that there was a sizeable community of South Asians like Asian labor migrants settled in European countries. Currently, approximately two million South Asians are living in Africa; some came in late nineteen and early twentieth century [47]. The phylogenetic analysis depicts that viral genome underwent various significant changes with time at different rates in which core region is considered to be more diverse [48]. A limited migration pattern has been identified among strains of Europe, North America, South America, and Africa that have shown high diversity in their respective geographical regions as reported previously that in areas of endemicity a highly divergent pattern is observed among the strains suggesting long infection duration [37]. It has been seen that core region is undergoing diversification at high rate in European, Asian, and South and North American countries. Some strains showing no branching in clade 2 of the tree giving an idea about probable “no change” occurrence among them for years as they were under high negative selection pressure due to some environmental factors. Clade 2, cluster VII showed some ancient HCV genotype 3 that might be circulating in suburban population of Rawalpindi. Clade 1 in the tree showed a clear independent cluster of sequenced samples; early branching in the tree at level 1 clearly indicates to a history of viral evolution that is very ancient in Pakistan as compared to other parts of the world [30][49]. Pybus et al. [37] explained the origin of HCV diversity and reported that Asian model of evolution could be a baseline for investigating HCV spread in other continents. Clade 1 has shown active and early branching pattern of subtype 3b in Pakistani population depicted that genotype 3 is actively evolving in suburban population of Pakistan.

### 5.2. Tajima’s Test of Neutrality

Test values indicated that the high mutation rate of the HCV might be one of the points of determinant action of the natural selection and thereby cooperated in inducing the divergence of viral species. At the beginning of HCV infection, there is a reduction in the viral population [50]. Despite positive values (D = 0.6568), the test indicates high levels of polymorphism and reduced population size thereby mediating a balancing selection process [51].

### 5.3. 3D Protein Structure Evaluation

Protein models were predicted using i-TASSER of the studied sequences. These 3D structures would be helpful in predicting the cleavage sites or recognition of phosphorylation sites. After translation is completed, during HCV replication, HCV polyprotein gets cleaved into at least ten distinct products. The order in which cleavages occur from N-terminus to C-terminus is - C-E1-E2-p7-NS2-NS3-NS4A-NS4B-NS5A-NS5B [52]. These protein help virus to maintain its structural integrity and protection against its host as well as incorporate virulence and pathogenesis to the virus such as envelop proteins. Interaction of some phosphorylation sites with kinases might be responsible for HCV resistance to antiviral effects of IFN which could be confirmed by analyzing these sites for different HCV genotypes [53]. With the help of predicted protein models the cleavage and phosphorylation sites in HCV polyproteins can be predicted and further targeted for designing an appropriate drug against resistant strains.

### 5.4. siRNA Prediction for HCV Core

The siRNA was predicted against five HCV samples that were resolved in the present study. Different domains of core perform different functions like siRNA predicted against all the genotypes (3a, 4a and 3b) positioned at N-terminal from 13-35 nucleotides contains RNA and DNA binding domain [1][2][3][4][5][6][7][8][9][10][11][12][13][14][15][16][17][18][19][20][21][22][23][24][25][26][27][28][29][30][31][32][33][34][35][36][37][38][39][40][41][42][43][44][45][46][47][48][49][50][54]. N- Terminal of core induces apoptosis and necrosis higher than those of C-terminal [55]. The results in this study demonstrated that siRNAs directed against domains (N-terminal and C-terminal) of HCV-3a Core gene and resulted in specific inhibition of HCV RNA synthesis (60-80 %) [56]. siRNAs targeted against HCV structural genes efficiently make full length HCV particles silent and provide an effective therapeutic option against HCV infection [57]. siRNA predicted against HCV core are from the same domain of core region showed that RNA and DNA binding domain was conserved in core region in genotype 3 and 4; so single siRNA can be used against both genotypes and inhibit HCV RNA synthesis. Multiple genotypes of HCV have been isolated throughout the world. The identification of HCV types and subtypes has major implications for HCV vaccine development. Characterization of these genetic groups is likely to facilitate and contribute to the development of an effective vaccine against infection with HCV. Currently, in addition to HCV genotype 3 (3a and 3b), two new genotypes have been reported for the first time: 4a from Rawalpindi and 6e from Pakistan. As high genetic diversity is shown throughout the world by phylogenetic analysis, a universally protected vaccine requires the addition of genotype-specific epitopes. Herein, little effort has been put in to design siRNA against the resolved genotypes of the study samples.
